# Ultra-sensitive in-situ detection of near-infrared persistent luminescent tracer nanoagents in crude oil-water mixtures

**DOI:** 10.1038/srep27993

**Published:** 2016-06-15

**Authors:** Yen-Jun Chuang, Feng Liu, Wei Wang, Mazen Y. Kanj, Martin E. Poitzsch, Zhengwei Pan

**Affiliations:** 1 College of Engineering, University of Georgia, Athens, GA 30602, USA.; 2 Department of Physics and Astronomy, University of Georgia, Athens, GA 30602, USA; 3 Aramco Research Center–Boston, Aramco Services Company, 400 Technology Square, Cambridge, Massachusetts 02139, USA; 4 Exploration and Petroleum Engineering Center–Advanced Research Center (EXPEC-ARC), Saudi Aramco, Dhahran, Saudi Arabia

## Abstract

Current fluorescent nanoparticles-based tracer sensing techniques for oilfield applications suffer from insufficient sensitivity, with the tracer detection limit typically at the several hundred ppm level in untreated oil/water mixtures, which is mainly caused by the interference of the background fluorescence from the organic residues in crude oil under constant external excitation. Here we report the use of a persistent luminescence phenomenon, which enables an external excitation-free and thus background fluorescence-free measurement condition, for ultrahigh-sensitivity crude oil sensing. By using LiGa_5_O_8_:Cr^3+^ near-infrared persistent luminescent nanoparticles as a tracer nanoagent, we achieved a tracer detection limit at the single-digit ppb level (down to 1 ppb concentration of nanoparticles) in high oil fraction (up to 65 wt.%) oil/water mixtures via a convenient, CCD camera-based imaging technique without any pretreatment or phase separation of the fluid samples. This detection limit is about four to five orders of magnitude lower than that obtained using conventional spectral methods. This study introduces a new type of tracer nanoagents and a new detection method for water tracer sensing in oil reservoir characterization and management.

Oil remains the most important and reliable source for world energy. Despite the great advancements made in oil operations, oil recovery is still less than optimal in the majority of oil fields: 50–75% of the oil in place cannot be recovered by conventional production methods^1^. To achieve higher oil recovery efficiency, it is necessary to properly characterize the reservoir and its fluid pathways using for example specially designed reservoir tracers. Two families of tracers, i.e., radioactive tracers^2^,^3^,^4^,^5^ and chemical tracers (e.g., fluorobenzoic acids)^5^,^6^,^7^,^8^, have been used and proven to be useful agents in gathering reservoir information. However, the deployment of these traditional tracers has become more restricted and limited due to either the increasing environmental concerns^5^ or the need for expensive and inconvenient analytic techniques (e.g., GC-MS and GC-MS/MS)^5^,^8^. Therefore, it is necessary to develop new types of tracers that are non-radioactive, can be conveniently analyzed in standard labs or on-site, and are chemically and physically stable in hostile (e.g., high temperature, high salinity, high pressure and corrosive) reservoir environments.

In the effort to find alternatives to traditional tracers, luminescent nanoparticles, such as carbon-based fluorescent nanoparticles^9^,^10^ and rare-earth ion doped nanoparticles^5^,^11^, have been investigated in recent years. Luminescent nanoparticles are expected to have a similar behavior to molecular tracers^12^,^13^ (but with lesser diffusivity in the rock matrix), and their unique optical properties can be analyzed by relatively simple or even portable spectral instruments. Although these optical nanoparticles exhibit certain promising characteristics, they suffer from a common drawback – an unsatisfactory detection sensitivity in the presence of crude oil, which is mainly caused by the strong autofluorescence from the highly fluorescent organic residues (e.g., polycyclic aromatic hydrocarbons) contained in crude oils under constant external excitation^14^. To suppress the background fluorescence, long fluorescence life-time tracer nanoagents, such as Au/fluorophore/silica fluorescent nanobeads^15^, were developed, allowing detection via time-resolved fluorescence spectroscopy. However, the reported detection remains poor – the tracer detection limit is about 200 ppm in synthetic seawater containing 5 wt.% crude oil^15^.

We recently developed a new type of luminescent nanoparticles – LiGa_5_O_8_:Cr^3+^ (LGO:Cr) near infrared (NIR) persistent luminescence nanoparticles with sizes of 50–100 nm^16^. The LGO:Cr nanoparticles emit strong photoluminescence at 716 nm under ultraviolet (UV) light excitation and long persistent luminescence (also called afterglow) after cessation of the excitation. During the UV excitation, the energy is stored as captured electrons in traps in the material. After the excitation ceases, the trapped electrons are gradually released and recombine with the ionized Cr^3+^ ions, followed by persistent emission at 716 nm of Cr^3+^ (ref. 16). Recently, the LGO:Cr nanoparticles were proven to be very promising optical nanoprobes for ultrasensitive deep-tissue biomedical imaging^17^. This is attributed to their persistent luminescence capability, which enables an external excitation-free and hence tissue autofluorescence-free imaging condition. The high detection sensitivity of this persistent luminescence-based biomedical imaging inspires us to apply persistent luminescence to the water tracer application in oilfield reservoir characterization and reservoir management operations. This is because the current fluorescence-based oilfield water tracer sensing techniques suffer from the same problems as the fluorescence-based biomedical imaging techniques, i.e., strong background autofluorescence and suboptimal detection sensitivity. Here, we report the first use of persistent luminescence in water tracer sensing. We demonstrate that, by taking advantage of the NIR persistent luminescence capability of LGO:Cr nanoparticles, an ultrahigh water tracer detection, with a detection limit well in the single-digit ppb level (down to 1 ppb), is achieved in oil/water emulsion samples containing up to 65 wt.% oil using a convenient CCD camera-based imaging technique.

## Results

Typical unrefined light crude oil from Saudi Arabia was used as received in our experiment. Certain amounts of crude oil (35, 50 and 65 wt.%), water and LGO:Cr nanoparticles were mixed to form homogeneous emulsions using an ultrasonic homogenizer. The emulsions were placed in 3.5 mL, 10 mm path-length UV-grade quartz cuvettes for measurements.

### Spectral method

**Figure 1:**
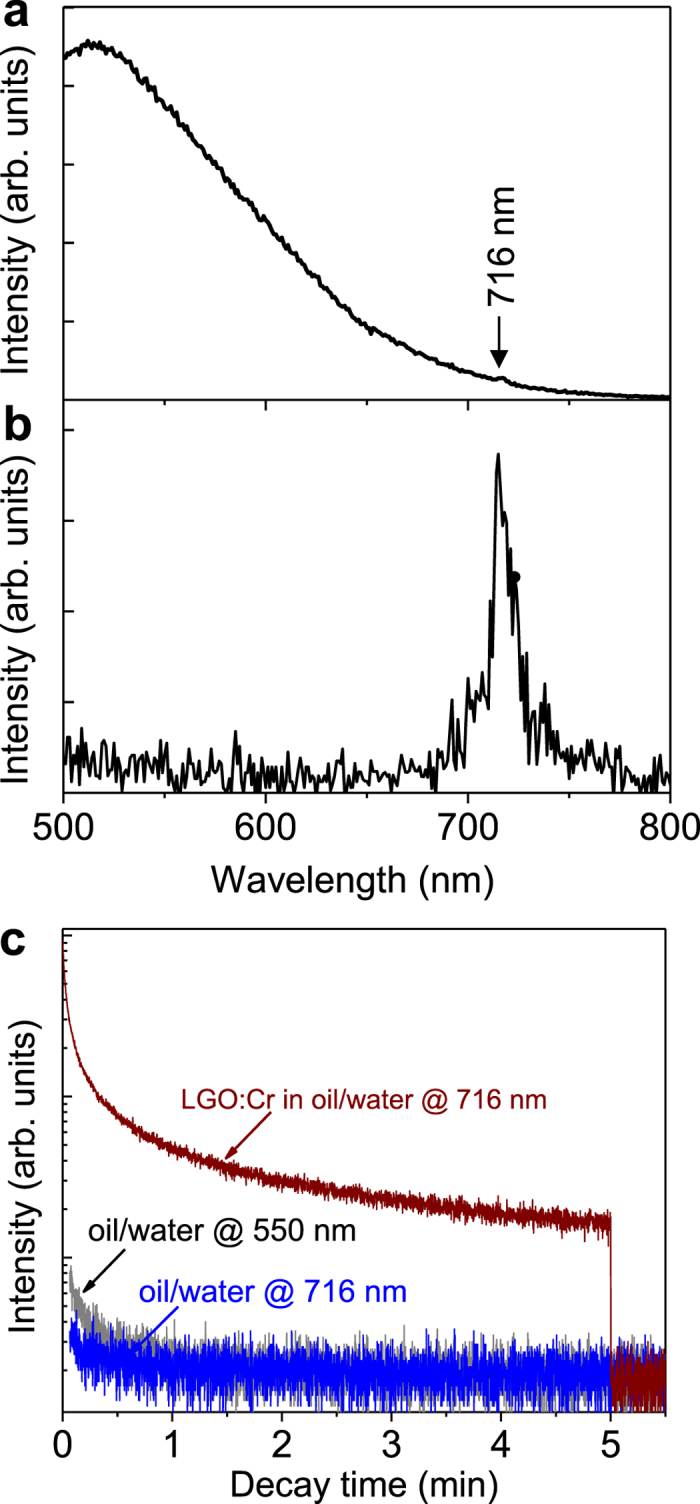
Spectral detection of 1,000 ppm LGO:Cr nanoparticles in 50/50 crude oil/water emulsions. (**a**) Photoluminescence emission spectrum under 254 nm UV light excitation. (**b**) NIR persistent luminescence emission spectrum recorded at 1 min after ceasing the UV irradiation. (**c**) Persistent luminescence decay curves of oil/water emulsions with and without LGO:Cr nanoparticles. The brown line curve was recorded by monitoring the 716 nm emission of an emulsion containing LGO:Cr nanoparticles. The acquisition of the 716 nm decay lasted for 5 min, after which, only the background signal was recorded. The grey and blue line curves were recorded by monitoring at 550 nm and 716 nm emissions, respectively, of an emulsion without LGO:Cr nanoparticles. The emulsions were irradiated by a 254 nm lamp for 5 min.

We first used a spectrofluorometer to detect the LGO:Cr nanoparticles in crude oil/water emulsions. Under the excitation of 254 nm UV light, the oil compounds emit a strong, broad photoluminescence band peaking at ~515 nm. The emission from the oil compounds is so strong that the NIR photoluminescence (peaking at 716 nm) from as high as 1,000 ppm LGO:Cr nanoparticles in 50/50 (weight ratio) oil/water emulsion is almost completely masked by it, as shown in the photoluminescence emission spectrum in Fig. 1a. However, when the excitation is ceased, the photoluminescence signal from the oil compounds disappears, but the NIR persistent luminescence signal from the LGO:Cr nanoparticles emerges (Fig. 1b), which can be readily detected by the spectrofluorometer. The persistent luminescence signal from the 1,000 ppm LGO:Cr nanoparticles in 50/50 crude oil/water emulsion can be clearly recorded for more than 5 min, as shown in the afterglow decay curve (brown line curve) monitoring at 716 nm emission in Fig. 1c. For comparison, Fig. 1c also shows the decay curves of a simple 50/50 crude oil/water emulsion monitoring at 550 nm emission (grey line curve) and at 716 nm emission (blue line curve). It is noticed from Fig. 1c that the oil compounds also exhibit persistent luminescence after ceasing the excitation, but it is weak and lasts less than 30 s. This weak and short afterglow from the oil compounds suggests that in order to completely eliminate the fluorescence from the crude oil, it is necessary to acquire the afterglow signal from the LGO:Cr nanoparticles with a delay time longer than the afterglow time of the oil compounds, such as 1 min, after stopping the excitation.

### Imaging method

The spectral results in Fig. 1 clearly show the advantage of persistent luminescence over photoluminescence in oilfield water tracer sensing. However, due to the low sensitivity of the spectrofluorometer to the inherently weak afterglow signals of the tiny amount of LGO:Cr nanoparticles in oil/water emulsions, the detection limit obtained by the spectral method is still considerably high, which is above 100 ppm. To take full advantage of the background interference-free condition, we then used a CCD camera-based imaging method, where an IVIS Lumina II imaging system, which is widely used in biomedical imaging^17^,^18^,^19^, was employed to collect the NIR afterglow signal and to take NIR afterglow images. In the imaging experiments, two quartz cuvettes, one containing the test sample (with LGO:Cr nanoparticles) and one containing the control sample (without LGO:Cr nanoparticles), were placed into the light-tight chamber of the IVIS imaging system at fixed positions. The samples were irradiated with a 254 nm UV lamp for 5 min. The images were taken 1 min after the cessation of the excitation with an exposure time of 5 min in the “bioluminescence” mode. By setting a 1 min delay, the background fluorescence, including the afterglow from the oil compounds, can be mostly eliminated, creating an imaging condition with minimum background interference. Therefore, using the IVIS-based imaging method, we are able to achieve a high water tracer detection sensitivity with the detection limit at the single-digit ppb level.

**Figure 2:**
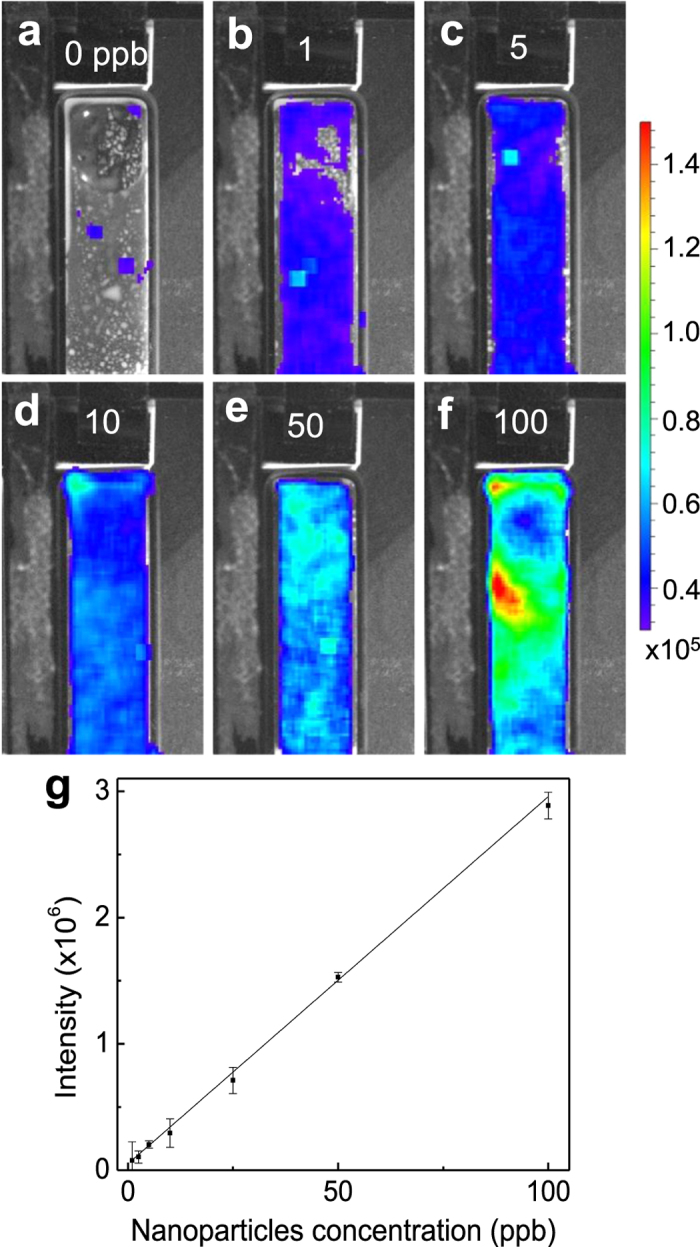
Detection of LGO:Cr nanoparticles in crude oil/water emulsions using an IVIS Lumina imaging system. (**a–f**) NIR afterglow images of 50/50 (weight ratio) oil/water emulsions containing 0, 1, 5, 10, 50 and 100 ppb LGO:Cr nanoparticles. The unit of the scale bar (photon radiance) is photon·second^−1^·cm^−2^·steradian^−1^ (p·s^−1^·cm^−2^·sr^−1^). (**g**) Plot of integrated afterglow intensity in (**a–f**) as a function of the concentration of LGO:Cr nanoparticles.

Figure 2a–f shows the NIR afterglow images of six 50/50 oil/water emulsions containing 0, 1, 5, 10, 50 and 100 ppb LGO:Cr nanoparticles. The images were acquired in photon radiance mode, and the afterglow intensity was presented as a color scale bar. The emulsion without LGO:Cr nanoparticles (Fig. 2a) was used as the control sample to quantify the background noise from oil compounds. It is clear that afterglow signals from as low as 1 ppb LGO:Cr nanoparticles in 50/50 oil/water emulsion can be clearly visualized across the entire top surface of the quartz cuvette (Fig. 2b), and the afterglow “brightness” increases as the amount of nanoparticles in the emulsions increases (Fig. 2b–f). This single-digit ppb level of detection limit is about five orders of magnitude better than that obtained using the spectral method by us (see Fig. 1) and others^15^. Based on the afterglow data for Fig. 2a–f and using the control sample (Fig. 2a) as the background, we calculated the integrated afterglow intensities from the different emulsions and plotted the intensity as a function of LGO:Cr nanoparticles concentration, as shown in Fig. 2g. As expected, the integrated afterglow intensity exhibits a linear relationship with the concentration of LGO:Cr nanoparticles in the emulsions.

**Figure 3:**
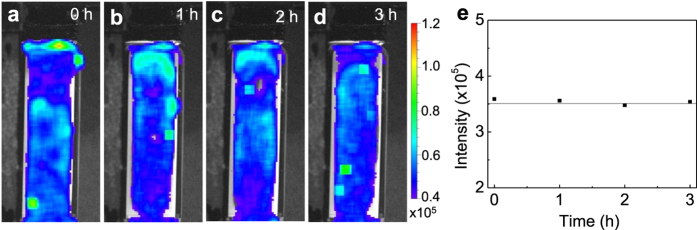
Colloidal stability and luminescence stability of a 50/50 crude oil/water emulsion containing 10 ppb LGO:Cr nanoparticles. (**a–d**) NIR afterglow images taken at 0, 1, 2 and 3 h after the emulsion was formed. The emulsion was irradiated with a 254 nm UV lamp for 5 min at every 1 h. (**e**) Plot of integrated afterglow intensity in (**a–d**) as a function of time.

The NIR images in Fig. 2b–f also show that the afterglow signals shine in the entire cuvettes, revealing that the LGO:Cr nanoparticles are uniformly dispersed in the oil/water emulsions. Such uniform dispersion can be stable for a long time even though no special surface treatments were made on the nanoparticles. Figure 3a–d shows the NIR afterglow images of a 50/50 oil/water emulsion containing 10 ppb LGO:Cr nanoparticles taken within 3 h after the emulsion was formed. The emulsion was irradiated with a 254 nm UV lamp for 5 min every 1 hour, and the image was taken 1 min after the stopping of each irradiation. No apparent change in the dispersion of the nanoparticles in the emulsion was observed within 3 h after the emulsion was formed, so as to the integrated afterglow intensity (Fig. 3e).

**Figure 4:**
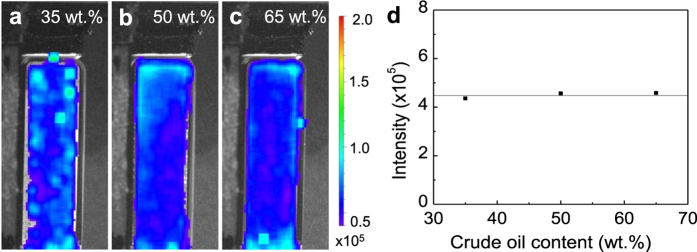
Effect of crude oil concentrations on the detection of LGO:Cr nanoparticles. (**a–c**) NIR afterglow images of 10 ppb LGO:Cr nanoparticles in 35/65, 50/50 and 65/35 crude oil/water emulsions. (**d**) Plot of integrated afterglow intensity in (**a–c**) as a function of the crude oil content in oil/water emulsions.

We also studied the effect of crude oil concentrations on the detection sensitivity of LGO:Cr nanoparticles in oil/water emulsions. Figure 4a–c shows the NIR afterglow images of 10 ppb LGO:Cr nanoparticles in 35/65, 50/50 and 65/35 oil/water emulsions. The results show that the amount of crude oil, even at a very high concentration of 65 wt.%, has no observable effects on the detection of the LGO:Cr nanoparticles. The integrated NIR afterglow intensities from the 10 ppb LGO:Cr nanoparticles in these three different oil/water emulsions are almost the same (Fig. 4d).

## Discussion

We have, for the first time, applied the persistent luminescence phenomenon in water tracer sensing and achieved a single-digit ppb level detection of tracer nanoparticles in high crude oil containing oil/water emulsions. This ultrahigh tracer detection capability relied on the use of LGO:Cr NIR persistent luminescent nanoparticles as the tracer material and a CCD camera-based IVIS imaging system as the detection tool. Since no *in-situ* external excitation is used during detection, the background fluorescence noise from the oil compounds, which is the major restriction of current fluorescence-based water tracer sensing, is mostly eliminated. Moreover, the persistent luminescence-based water tracer sensing avoids the need of sophisticated oil-water phase separation procedure as well as the need of expensive and inconvenient analytic tools. The potential for real-time, ultrahigh sensitive detection along with the excellent chemical and thermal stability of the material^16^, make the persistent luminescence-based sensing technique very promising for application in oil reservoir characterization, monitoring and management. Moreover, the persistent luminescence-based sensing technique is also expected to have impacts on other applications (e.g., biomedicine and pharmaceuticals) where highly sensitive, reliable and convenient detection is needed.

## Methods

### Preparation of crude oil/water/LiGa_5_O_8_:Cr^3+^ (LGO:Cr) nanoparticles emulsions

Unrefined crude light oil from Saudi Arabia was used without any pretreatment. Certain amounts of crude oil, water and LGO:Cr nanoparticles were mixed to form homogeneous emulsions via an ultrasonic homogenizer (Omni Sonic Ruptor 400). The oil content in the emulsions varied at 35 wt.%, 50 wt.% and 65 wt.%. The emulsions were placed in 3.5 mL, 10 mm path-length UV-grade quartz cuvettes for measurements.

### Characterization methods

Both a spectral method and a CCD camera-based imaging method were used to acquire the NIR signals from the LGO:Cr nanoparticles in crude oil/water emulsions. The spectral measurements were conducted on a Horiba FluoroLog-3 spectrofluorometer equipped with a 450 W xenon arc lamp and a R928P photomultiplier. The acquired spectral data included photoluminescence emission spectra, persistent luminescence emission spectra and persistent luminescence decay curves. For the decay measurements, a 254 nm UV lamp was used to excite the samples for 5 min. Appropriate optical filters were used to avoid stray light in all spectral measurements.

The imaging experiments were performed on an IVIS Lumina II imaging system in the bioluminescence mode. The quartz cuvettes containing crude oil/water/LGO:Cr nanoparticles emulsion were placed inside the imaging chamber at fixed positions. The samples were irradiated with a 254 nm UV lamps for 5 min and the images were taken 1 min after the stoppage of the excitation with an exposure time of 5 min. Each set of imaging measurement was repeated at least three times. The obtained images were processed using Living Image software (Version 4.4 SP2, PerkinElmer) at a binning of 8 and smoothing of 5x5.

## Additional Information

**How to cite this article**: Chuang, Y.-J. *et al.* Ultra-sensitive in-situ detection of near-infrared persistent luminescent tracer nanoagents in crude oil-water mixtures. *Sci. Rep.* **6**, 27993; doi: 10.1038/srep27993 (2016).
